# Importance of blood pressure lowering in patients with direct oral anticoagulant-associated intracerebral haemorrhage in the acute phase and for secondary prevention

**DOI:** 10.1177/23969873231208544

**Published:** 2025-05-22

**Authors:** Adrian R Parry-Jones, Tom J Moullaali, Else C Sandset, Adnan I Qureshi, Craig S Anderson, Thorsten Steiner

**Affiliations:** 1 Geoffrey Jefferson Brain Research Centre, Manchester Academic Health Science Centre, Northern Care Alliance & University of Manchester, Manchester, UK; 2 Division of Cardiovascular Sciences, Faculty of Biology, Medicine and Health, University of Manchester, Manchester, UK; 3 Centre for Clinical Brain Sciences, University of Edinburgh, Edinburgh, UK; 4 Department of Clinical Neurosciences, NHS Lothian, Edinburgh, UK; 5 Department of Neurology, Stroke Unit, Oslo University Hospital, Oslo, Norway; 6 The Norwegian Air Ambulance Foundation, Oslo, Norway; 7 Zeenat Qureshi Stroke Institute and Department of Neurology, University of Missouri, Columbia, MO, USA; 8 The George Institute for Global Health, University of New South Wales, Sydney, NSW, Australia; 9 The George Institute China, Beijing, P.R. China; 10 Neurology Department, Royal Prince Alfred Hospital, Sydney Health Partners, Sydney, NSW, Australia; 11 Departments of Neurology, Klinikum Frankfurt Höchst and Heidelberg University Hospital, Frankfurt, Germany

**Keywords:** Intracerebral haemorrhage, direct oral anticoagulants, blood pressure

## Abstract

**Purpose:**

Intracerebral haemorrhage (ICH) is an important complication of direct oral anticoagulation (DOAC) therapy, where risks and prognosis are potentially modified by effective blood pressure (BP) control, both in the acute phase and for secondary prevention. Herein, we review BP management in the context of general anticoagulation associated ICH and specifically in DOAC-ICH, considering current evidence and highlighting outstanding questions.

**Method:**

Narrative review.

**Findings:**

Pooled analyses of major trials of BP lowering in acute ICH patients without anticoagulants demonstrate a reduction in the risk of haematoma expansion. As anticoagulant-associated ICH patients tend to be older, have more co-morbidities, and larger haematomas at baseline with a greater risk of expansion, the risks and benefits of intensive BP lowering treatment might both be higher. Small observational studies of DOAC-ICH patients suggest that lower achieved BP is associated with less expansion, lower mortality, and better functional outcomes. Care bundles including both anticoagulant reversal and intensive BP lowering might reduce the risk of death and disability in DOAC-ICH. Optimal control of BP in survivors of ICH reduces the risk of both ischaemic and haemorrhagic stroke but whether this modulates the risks and benefits of restarting a DOAC is unknown.

**Discussion:**

Limited evidence suggests that BP should be well managed in DOAC-ICH patients, in the same way as ICH patients not on anticoagulants, both in the hyperacute phase and for secondary prevention. Hypothetical differences in the effects of BP lowering treatment in DOAC-ICH need to be tested in clinical trials.

## Introduction

**Figure 1.**
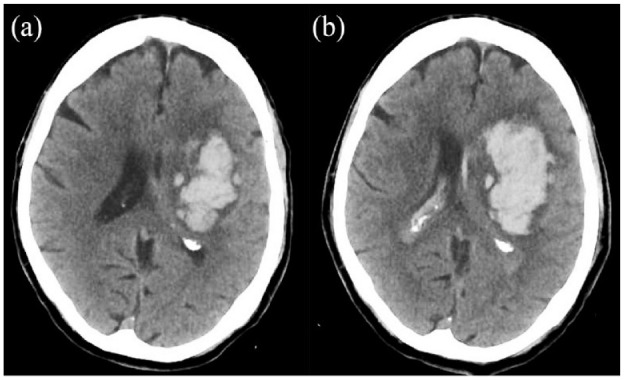
CT brain scans from a patient taking rivaroxaban for atrial fibrillation and a blood pressure on presentation of 183/94. The baseline scan: (a) was 2 h after symptom onset and the second scan and (b) was completed 3 h later. Within 1 h of the baseline scan, systolic blood pressure was lowered to <140 mmHg and 50 IU/kg of four-factor prothrombin complex concentrate was administered. Despite treatment, the haematoma expanded from 52.8 ml (scan a) to 83.1 ml (scan b), an expansion of 30.3 ml (57%).

Anticoagulation is increasingly used to reduce the risk of stroke in patients with atrial fibrillation (AF) as well as other indications such as venous thromboembolism and prosthetic heart valves. Direct oral anticoagulants (DOACs) have been available for nearly two decades and are now the most widely used anticoagulant for patients with AF in Western Europe and the US.^1^ Intracerebral haemorrhage (ICH) is the most serious complication of anticoagulation, but all of the major DOAC trials demonstrate a lower risk of ICH compared to traditional, vitamin K antagonist (VKA) anticoagulation.^2–5^ Despite the promise of lower ICH rates overall with DOACs including those aged 85 and older,^6^ anticoagulant-associated ICH continues to present a major challenge to clinicians as a greater proportion of elderly patients with higher risk of ICH are anticoagulated. Current ICH treatment is aimed at reducing haematoma expansion (Figure 1), the risk of which is determined by time from onset to presentation, haematoma volume at baseline and antithrombotic treatment.^7^ Imaging markers such as the spot-sign on computed tomography (CT) angiography source images^7^ and the shape and heterogeneity of the haematoma on non-contrast CT^8^ can also help to stratify risk. ICH is also characterised by a cascade of secondary injury, which is worsened when haematoma expansion occurs.^9^ Evidence regarding reversal strategies to reduce the risk of haematoma expansion in DOAC-ICH has struggled to keep pace with prescribing patterns, with initial use of prothrombin complex concentrate (PCC) gradually being replaced by specific reversal agents.^10,11^ As DOACs were becoming widely used, evidence from the main INTEnsive blood pressure lowering in Acute Cerebral haemorrhage Trial (INTERACT2)^12^ led to intensive blood pressure (BP) lowering being recommended in guidelines.^13^ Anticoagulant reversal and intensive BP lowering have subsequently become the key components of acute care bundles for DOAC-associated ICH.^14^ Beyond the acute phase, optimal management of hypertension in the months to years after DOAC-ICH is vital for the prevention of both ischaemic stroke and recurrent ICH.^15^ Uncontrolled high BP, which is the strongest modifiable risk factor of ICH,^16^ and features in bleeding risk scores such as HAS-BLED,^17^ may influence decisions about the resumption of DOACs due to perceived bleeding risks. Herein, we review evidence relevant to BP management in DOAC-ICH, firstly in the hyperacute phase and then part of secondary prevention.

## Hyperacute BP management in ICH

Data informing how hyperacute BP should be managed specifically in DOAC-ICH are scarce. Therefore, we first consider evidence from clinical trials of intensive BP management in ICH (which included few patients on anticoagulants) and consider choice of anti-hypertensives. We then review the limited evidence for BP management in DOAC-ICH as well as drawing on evidence from studies concerning ICH associated with all antithrombotics, all anticoagulants, or VKA-ICH specifically. We then consider whether hyperacute BP management should be different in DOAC-ICH and any opportunities for further research.

### Clinical trials of intensive BP lowering in ICH

A hypertensive response is common in the first 24 hours of ICH onset and is associated with haematoma expansion, perihaematomal oedema, death and disability.^18^ INTERACT2 demonstrated that intensive systolic BP (SBP) lowering (target <140 mmHg within 1 h) compared to guideline management (target <180 mmHg) did not significantly reduce the odds of death or disability (modified Rankin Scale [mRS] 3–6) at 3 months in the primary analysis but a prespecified secondary ordinal analysis showed significantly lower mRS with intensive treatment (odds ratio 0.87; 95% confidence interval, 0.77–1.00; *p* = 0.04).^12^ The generalisability of INTERACT2 to DOAC-ICH is limited by the inclusion of <3% of patients on anticoagulants (all taking warfarin) and the preponderance of deep (83%), small (median ICH volume of 11 ml) ICHs included. However, the parallel second Antihypertensive Treatment of Cerebral Haemorrhage (ATACH-2) trial where intensive (target SBP 110–139 mmHg) was compared to guideline (140–179 mmHg) SBP management with intravenous nicardipine as the primary antihypertensive agent, found no difference in death or disability at 90 days.^19^ ATACH-2 excluded patients with an INR > 1.4 or taking a DOAC and also showed an increase in renal adverse events in the intensive BP group. A pre-planned pooled analysis of INTERACT2 and the ATACH-2 trial demonstrated that lower achieved SBP (mean of SBP 1–24 h) and lower variability (SD of SBP 1–24 h) were both associated with better functional outcome and a lower risk of haematoma expansion.^20^ An individual participant data meta-analysis of 6221 patients from 16 trials reported that active/intensive BP-lowering interventions within 7 days had no effect on ordinal analysis of mRS compared with placebo/guideline treatment.^21^ Active/intensive BP-lowering interventions reduced absolute (>6 ml) and relative (⩾33%) haematoma expansion. Most guidelines recognise lowering SBP to a target range of 130–140 mm Hg as a safe intervention that likely improves functional outcomes in mild to moderate ICH within 6 h of onset and where the presenting SBP is 150–220 mmHg.^22,23^ Initiating treatment as soon as possible with careful titration of antihypertensive agents to ensure continuous, smooth, and sustained control of BP is generally recommended. However, the safety and efficacy of intensive SBP reduction in patients with very high SBP (>220 mmHg) and in those with large and more severe ICHs, requires further study as these patients were not adequately represented in these trials.

### Antihypertensive drugs for treatment of ICH

Early (<1 h) lowering of SBP seems to be associated with favourable outcomes,^20^ and patients treated intravenously, and especially with α- and β-adrenoreceptor blockers and calcium channel blockers, have better functional outcomes.^21^ These results define important requirements for antihypertensive drugs in the setting acute ICH: fast action, good controllability, minimal side-effects.^24^ Drugs that have been used in trials to lower BP in acute ICH are summarised in Table 1.^12,19,25–28^ A limited number of agents fulfil requirements for use in the setting of acute ICH, that is of an intravenous route and short onset time, for example, the alpha-1 agonist urapidil, beta-blockers like metoprolol or labetolol, the alpha-2 agonist clonidine, and calcium channel blockers like nicardipine or clevidipine. Of these, several have a very short half-life, including clevidipine, sodium nitroprusside, and glyceryl trinitrate. The latter agent, however, might have some deleterious effects in patients with acute ICH, at least in the prehospital setting,^29,30^ whereas nitroprusside is a strong vasodilator and associated with cyanate toxicity.^31^ Available data on clevidipine are scarce. It was administered to 35 patients with acute ICH within 6 and 12 h in a prospective open-label trial, with only minimal haematoma expansion being observed.^26^ A systematic review and meta-analysis comparing nicardipine and clevidipine came to the conclusion that SBP goals were reached faster with clevidipine, although this did not reach statistical significance.^32^ Clevidipine was also used in the recently announced INTEnsive care bundle with blood pressure Reduction in Acute Cerebral haemorrhage Trial (INTERACT3), but outcomes specifically in relation to different agents are not yet available.^14^

**Table 1.** Agents used for intensive BP lowering in ICH.

Agent	Half-Life	IV Use	Most important side effects
ACE inhibitors			
Captopril^73^	2 h	Y	Hyperkalaemia, dry cough, creatinine elevation in patients with chronic renal insufficiency or renal artery stenosis, rarely angioedema, contraindicated in pregnancy
Enalapril^73^	11 h	Y	As above
Ramipril^73^	13–17 h	Y	As above
Alpha-1 blockers			
Urapidil^74^	2.7 h	Y	Hypotension, reflex tachycardia
Alpha-2 agonists			
Clonidine^75^	15.8 h	Y	Dry mouth, drowsiness, bradycardia
Angiotensin-II receptor blockers			
Losartan^76^	1–4 h	N	Hypotension, hyperkalaemia, dizziness
Beta-blockers			
Atenolol^77^	6–9 h	N	Bradycardia, hypotension, fatigue, depression, sexual dysfunction
Carvedilol^77^	6–10 h	Y	Hypotension, bradycardia, fatigue
Esmolol^77–79^	9 min	Y	As above
Metoprolol^77^	3–4 h	Y	As above
Propranolol^77^	3–4 h	Y	As above
Labetalol^77^	5–8 h	Y	As above
Calcium channel blockers			
Amlodipine^80^	40–60 h	N	Peripheral oedema, headache, flushing
Clevedipine^81^	1.5 min	Y	Hypotension, nausea, vomiting, tachycardia, bradycardia
Nitrovasodilators			
Nitroprusside^82^	2 min	y	Headache, reflex tachycardia, flush, dizziness, severe hypotension
Glycerol trinitrate^83^ (nitrogylcerin)	<24 h	transdermal	Headache, reflex tachycardia, flush, dizziness, severe hypotension

### Intensive BP lowering in antithrombotic-associated ICH: Current evidence

Observational studies support the importance of BP control in reducing the risk of haematoma expansion in DOAC-ICH. A German study involving a retrospective cohort of 146 DOAC-ICH patients showed that achieving an SBP < 160 mmHg within 4 h of admission was significantly associated with a reduction in haematoma expansion, whilst administration of PCC was not.^33^ A previous study by the same investigators in 853 patients with VKA-ICH also showed that achieving both SBP < 160 mmHg and an international normalised ratio (INR) <1.3 within 4 h of admission were independently associated with lower rates of haematoma expansion.^34^

A Norwegian study explored interactions between antithrombotic use and prehospital BP in a cohort of 420 patients with spontaneous ICH. Sixty percent of all cases were taking an antithrombotic at presentation (22% were taking an anticoagulant, 33% were taking an antiplatelet drug and 5% were taking a combination of an antiplatelet drug and an anticoagulant).^35^ In patients on anticoagulant treatment (vs those not on an antithrombotic), a higher prehospital SBP was more strongly associated with larger haematoma volumes, higher risk of in-hospital mortality, and a greater detrimental shift in the mRS scores after adjustment for potential confounding factors. Patient characteristics were described for all patients on antithrombotics combined and not by subgroups. The combined antithrombotic group were more often men, older, had higher pre-ICH mRS scores, and more comorbidities (history of ischaemic stroke, coronary artery disease, diabetes, AF, and treated hypertension), suggesting a frailer population. At baseline, patients on antithrombotics had more lobar ICH, more intraventricular extension, and received more do-not-resuscitate orders in the first 24 hours. Furthermore, patients on antithrombotics had greater haematoma expansion (33%vs 18%, *p* = 0.026) and higher in-hospital (34%vs 18%, *p* < 0.001) and 90 days (44%vs 28%, *p* < 0.001) mortality.

There is little randomised controlled evidence for BP management in DOAC-ICH, but a subgroup analysis of the INTERACT studies, including 364 patients with antithrombotic-associated ICH (279 antiplatelet only, 71 anticoagulant only, 14 combination of both), found no heterogeneity in the effect of BP lowering on death or dependency by prior use of antithrombotics.^36^ Intensive BP lowering appeared to reduce haematoma expansion more in antithrombotic-treated patients than in those without (4.7vs 1.3 ml, *p* for interaction = 0.104). As seen in the Norwegian study, patients taking antithrombotics were frailer; they were older with more comorbidities and had lower baseline BP, and more lobar ICH and intraventricular extension. As part of a care bundle including anticoagulant reversal, intensive BP lowering reduced both death and disability in the large INTERACT3 study but <1% of patients were taking an anticoagulant, the great majority of whom took warfarin.^14^ A before versus after study of a similar care bundle where 14% of patients were on anticoagulants, including DOACs, demonstrated a 10.8 percentage point reduction in 30-day case-fatality.^37^

### Should BP in DOAC-ICH be managed differently?

We hypothesise that different characteristics of ICH in patients taking anticoagulants could impact upon the hyperacute management of BP. Much of what we know in relation to anticoagulant-associated ICH comes from studies undertaken prior to the introduction of DOACs. Compared to ICH patients not taking VKAs, VKA-ICH patients tend to be older (by a mean of 6 years^38^), have more co-morbidities (diabetes, ischaemic heart disease, previous ischaemic stroke) and unsurprisingly, more of the key indications for anticoagulation, including AF and prior venous thromboembolism.^39–41^ VKA-ICH is typically more severe, with baseline ICH volume approximately 10 ml higher,^2^ lower admission GCS, and an odds of haematoma expansion that is nearly three times greater than that for patients not taking anticoagulants.^38^ A small study has suggested that haematoma expansion may occur later on VKAs,^42^ but a subsequent meta-analysis describing a predictive model for haematoma expansion that included time from onset-to-scan reported similar discrimination in patients on anticoagulants compared to those who were not.^7^ In regard to haematoma location, a population-based study in France investigating time trends in ICH characteristics from 1985 to 2008 showed an increase in older patients (>75 years) with lobar ICH who are also taking anticoagulants.^43^ Other data regarding location of ICH and VKAs are conflicting, with three small studies suggesting a higher incidence of infratentorial ICH with VKA,^40,41,44^ but three other studies not identifying such a relationship.^39,45,46^ A meta-analyses of studies comparing VKA-ICH to DOAC-ICH found that after adjustment for confounders, DOAC-ICH was associated with lower admission haematoma volume and stroke severity but no difference in the odds of haematoma expansion, death, or poor functional outcome.^47^ However, patients included in this meta-analysis were three times more likely to receive reversal for VKA than a DOAC, so the widespread use of DOAC reversal agents may have since selectively improved DOAC-ICH outcomes.

Collectively, these data demonstrate that anticoagulant-associated ICH patients tend to be older, have more co-morbidities (including ischaemic stroke and ischaemic heart disease), and larger haematomas (possibly more often in a lobar location) with a greater risk of expansion. Given larger baseline ICH volumes, more patients are likely to have raised intracranial pressure (ICP) and thus reduced cerebral perfusion pressure (CPP), potentially leading to an excessive reduction in CPP with intensive BP lowering. As with larger ICH volumes in patients not on anticoagulants, monitoring of ICP to provide a patient specific CPP target may be desirable. A higher risk of haematoma expansion in DOAC-ICH patients might mean greater potential for benefit of intensive BP lowering which reduced haematoma expansion in a large meta-analysis.^21^ However, the largest trials in the meta-analysis either included only small numbers of patients taking an anticoagulant (2-3% in the INTERACT trials) or excluded them (ATACH-2 excluded patients with an INR > 1.4 or taking a DOAC). One may also hypothesise that the treatment window for intensive BP lowering may be longer in DOAC-ICH, given some prior evidence that patients may remain at risk of haematoma expansion for longer when anticoagulated, and that given the relatively high incidence of co-morbidities in DOAC-ICH patients, they may be at higher risk of complications of intensive BP lowering, including acute kidney injury and cardiac ischaemia. Further research will be required to test these hypotheses.

### Opportunities for further research

Randomised clinical trials are the gold standard for producing unbiased evidence of treatment effects but clinical trials addressing outstanding questions regarding intensive BP lowering in DOAC-ICH will present major challenges. Even in countries with high use of anticoagulants, DOAC-ICH constitutes no more than 2-3% of stroke patients presenting to stroke services and patients may be very unwell on presentation. Furthermore, intensive BP lowering can be challenging to implement alongside other urgent treatments, taking a considerable amount of time and attention to implement effectively. Having sufficient funding and infrastructure for large-scale clinical trials to overcome these challenges will be vital. Recent developments and novel methods may provide some solutions to challenges and include the standardisation of trial protocols to facilitate comparisons and individual patient data (IPD) pooling projects; use of adaption and platform designs; probabilistic interpretations and Bayesian statistics; care bundle interventions with potential synergistic effects; and the integration of clinical trials within routine clinical practice. Given that INTERACT3 included very few patients on anticoagulants, further cluster-randomised trials of care bundles for ICH including anticoagulant reversal and intensive blood pressure lowering in healthcare systems with high anticoagulant use may help to address current uncertainties. Finally, prehospital SBP seems to be more strongly associated with worse outcomes in anticoagulant-associated ICH patients compared to those not on antithrombotics.^35^ Targetting prehospital SBP may thus be of benefit, but recent trials of glyceryl trinitrate patches in suspected stroke patients in the prehospital setting have suggested possible harm in patients with intracerebral haemorrhage,^29,30^ suggesting that alternative agents may be needed to target prehospital SBP in future trials, as mentioned above.

## Long-term BP management in DOAC-ICH patients restarted on DOACs

It is well established that good BP control can reduce the risk of first-ever and recurrent stroke; the lower the better, provided there are no contraindications.^48,49^ For survivors of ICH, BP-lowering can reduce the risk of recurrent stroke by 50%, and this is the mainstay of secondary prevention.^23,50,51^ Separately, anticoagulation has a powerful effect on reducing thromboembolic risk from AF, and is recommended in people with AF deemed at higher risk of stroke using the widely used CHA_2_DS_2_-VASc score.^52,53^ However, there is ongoing uncertainty about the best strategy for balancing the risk of future major ischaemic and haemorrhagic events after ICH with regards to antithrombotic medication, including use of DOACs in the context of comorbid AF as well as other indications, such as venous thromboembolism, where the balance of risks and benefits may differ. Achieving good control of BP might be especially important in survivors of DOAC-ICH, as the risk of recurrent stroke is high in this group; such people are likely to be at high risk of severe ischaemic stroke from AF ^54^; and may potentially be exposed to increased risk of severe recurrent ICH should their DOAC be restarted.^55^ Therefore, a secondary prevention strategy that balances risks of major ischaemia versus major haemorrhage is required with BP-lowering being a key component as it reduces the risk of both event types. For patients with venous thromboembolism, their risk of recurrent venous thrombosis must be carefully considered alongside the risk of haemorrhage.

The pursuit of a ‘personalised medicine’ approach requires robust data to guide clinicians over secondary prevention, where the potential risks and benefits vary across individuals. DOAC-ICH in the context of comorbid AF provides a paradigm to highlight challenges in this area where significant potential benefits and risks exist from various approaches. At the present time, there are few data to guide these decisions; this section aims to outline available data, ongoing research and future directions.

We hypothesise that achieving good BP control might be especially important in ICH survivors with comorbid AF who are prescribed a DOAC, because BP-lowering reduces the risk of major ischaemic cardiovascular events for which this group is at high risk as well as reducing the risk of ICH, which can be more severe when taking a DOAC. It is worth considering the balance of absolute risks of major ischaemic and haemorrhagic events after ICH in general. Pooled data from two population-based studies of ICH survivors^56^ reported an annual risk of recurrent ICH of around 3% and ischaemic stroke of around 2%, suggesting the risks are fairly comparable in the broadest sense. However, crude grouping of ICH survivors by comorbid AF status (present vs absent) and ICH location (lobar vs deep) showed some divergence in ischaemic and haemorrhagic risks: the annual risk of ischaemic stroke was highest in ICH survivors with comorbid AF (approx. 6%); the annual risk of bleeding was highest in ICH survivors with lobar ICH location (approx. 5%). Other factors may modify these risks, including use of DOACs, where data from small RCTs revealed the risk of recurrent ICH may be around double compared with ICH survivors who avoid them.^55^ Observational data show that the higher the BP during follow-up, the higher the risk of recurrent ICH, with a steep increase in risk associated from SBP levels above 130 mmHg.^57^ It is possible that high BP and DOAC use interact to confer additional risk over-and-above the risk posed individually, although research about risk factor clusters for recurrent major cardiovascular events after ICH are lacking. High BP is an established risk factor for AF, and it is possible that lowering BP may reduce AF burden and its associated risks. However, a recent large individual patient data meta-analysis did not reveal an interaction between the presence or absence of AF and the effect of BP-lowering on major cardiovascular events.^58^

Further research is required to assess the interaction between BP-lowering and DOACs after ICH. This issue may be especially important in people with higher bleeding risk, such as those with radiographic evidence of bleeding prone vasculopathy, for example, where multiple cerebral microbleeds are detected,^59^ or where imaging criteria for probable cerebral amyloid angiopathy (CAA) are met.^60^ Post-hoc analysis of the PROGRESS trial suggested BP lowering reduced the risk of probable CAA-related ICH after an index event of TIA, ischaemic stroke or ICH.^61^ Therefore a beneficial interaction is plausible and may mitigate some of the risk from DOAC use after ICH. An updated Cochrane review presented data from small RCTs investigating the effects of restarting versus avoiding anticoagulation after ICH, showing probable reductions in the risk of major cardiovascular events and all major occlusive vascular events, but no difference in death during follow-up, probably due to increased risk of fatal ICH.^55^ Data from an ongoing main-phase trial are required (NCT03950076), and pooled with several smaller RCTs^62–64^ may facilitate subgroup analyses to test the interaction between BP control and anticoagulant use during follow-up.

Essential of any personalised secondary prevention strategy after ICH is its safety, acceptability and tolerability, and its uptake by clinicians and ICH survivors. There are no data to suggest long-term BP-lowering in survivors of DOAC-associated ICH might be less safe, acceptable or well-tolerated than in people with ICH not associated with DOACs, although the risk of falls from postural hypotension may be considered when deciding whether to restart a DOAC. Research about concordance with guideline-recommended secondary prevention strategies shows that uptake could be substantially improved.^65–67^ Qualitative research has revealed patient and medication-related barriers to adherence to secondary prevention may exist, including difficulty taking medication, beliefs about medication and the complexity of treatment regimens.^68^ We need simple, effective BP-lowering regimes for people with DOAC-associated ICH and data to guide the selection of patients suitable for restarting of DOACs.

The Triple Therapy Prevention of Recurrent Intracerebral Disease Events Trial (TRIDENT) aims to recruit 1500 ICH survivors and will report the effects of a novel BP-lowering ‘triple pill’ on the rate of any stroke during follow-up. The combination pill approach has been shown to be associated with larger BP reductions^69^ and better self-reported adherence,^70^ but its effect on outcomes after stroke remains uncertain for the time being. There may be scope to assess heterogeneity of effect by antithrombotic use during follow-up, but patient numbers are likely to be small given ongoing uncertainty about the best approach to managing the risk of major vaso-occlusive events in ICH survivors. Once we have data to guide decisions about DOAC use after ICH, the next step might involve testing combination pills which include an antithrombotic, following on from trials that have shown this approach to be effective for primary prevention^71^ and secondary prevention after myocardial infarction.^72^

## Summary and conclusions

DOAC-ICHs now represent the majority of anticoagulant-associated ICH cases in Western Europe and North America. Whilst our narrative review has limitations relative to a systematic review of the literature, we have found that relative to ICH patients not taking anticoagulants, DOAC-ICHs occur in older patients with more co-morbidities, haematomas are larger at baseline and are more likely to expand. BP lowering reduces haematoma expansion in patients not taking anticoagulants, limited observational evidence suggests that DOAC-ICH benefits benefit at least as much, and care bundles including anticoagulant reversal and BP lowering reduce death and disability. Uncertainties remain regarding heterogeneity of the benefits and risks of intensive BP lowering due to baseline differences and disease severity of acute DOAC-ICH. Future research should seek to address this. Survivors of DOAC-ICH have their risk of both recurrent ICH and ischaemic stroke reduced by optimal BP control, and this may modulate the risk and benefits of restarting DOACs, especially in patients with underlying AF. Ongoing trials of avoiding versus restarting anticoagulants for AF after ICH should help to further shed light on this important area. In the meantime, clinicians should manage BP in DOAC-ICH the same way as patients not taking an anticoagulant, as part of an acute bundle of care for all ICH patients, quickly (within 1 h) lowering SBP to a target of <140 mmHg in patients presenting within 6 h of onset with a SBP between 150 and 220 mmHg and maintaining smooth and ongoing control for the next 7 days, as recommended in current guidelines.^23^
